# Tree Height Growth Measurement with Single-Scan Airborne, Static Terrestrial and Mobile Laser Scanning

**DOI:** 10.3390/s120912798

**Published:** 2012-09-19

**Authors:** Yi Lin, Juha Hyyppä, Antero Kukko, Anttoni Jaakkola, Harri Kaartinen

**Affiliations:** Finnish Geodetic Institute, Geodeetinrinne 2, 02430 Masala, Finland; E-Mails: juha.hyyppa@fgi.fi (J.H.); antero.kukko@fgi.fi (A.K.); anttoni.jaakkola@fgi.fi (A.J.); harri.kaartinen@fgi.fi (H.K.)

**Keywords:** tree growth surveying, airborne, static terrestrial, mobile, laser scanning

## Abstract

This study explores the feasibility of applying single-scan airborne, static terrestrial and mobile laser scanning for improving the accuracy of tree height growth measurement. Specifically, compared to the traditional works on forest growth inventory with airborne laser scanning, two issues are regarded: “Can the new technique characterize the height growth for each individual tree?” and “Can this technique refine the minimum growth-discernable temporal interval further?” To solve these two puzzles, the sampling principles of the three laser scanning modes were first examined, and their error sources against the task of tree-top capturing were also analyzed. Next, the three-year growths of 58 Nordic maple trees (*Crimson King*) for test were intermittently surveyed with one type of laser scanning each time and then analyzed by statistics. The evaluations show that the height growth of each individual tree still cannot be reliably characterized even by single-scan terrestrial laser scanning, and statistical analysis is necessary in this scenario. After Gaussian regression, it is found that the minimum temporal interval with distinguishable tree height growths can be refined into one month based on terrestrial laser scanning, far better than the two years deduced in the previous works based on airborne laser scanning. The associated mean growth was detected to be about 0.12 m. Moreover, the parameter of tree height generally under-estimated by airborne and even mobile laser scanning can be relatively revised by means of introducing static terrestrial laser scanning data. Overall, the effectiveness of the proposed technique is primarily validated.

## 1. Introduction

The knowledge of tree height growths is increasingly required in a variety of domains, which range from forest harvest prediction for land-use planning [1] to forest dynamics characterization for climate anomaly response [2]. Consequently, the techniques for accurate measurements of tree height growths are highlighted. The traditional photogrammetric methods have already been used for long-term forest height growth surveys. Their common strategy is to reconstruct the associated canopy surface models from the stereo-pairs of aerial images, and the height differences between such two models relative to different time, e.g., 32 years apart [3] or even 58 years apart [4], are deemed as the height growths of the forests of interest. However, this theme applicable for tree height growth measurement is briefly valid for very flat lands or open forests. As suggested by Vanclay [5], new measurement techniques need to be explored for effective tree height growth monitoring.

Apart from further enhancement of the efficiencies of the photogrammetric methods, laser scanning proved to be another effective plan [6]. Since 1990s, airborne laser scanning (ALS) has been widely exploited in various forest-relevant researches and applications [7]. The previous attempts of applying ALS for forest inventories, including directly for tree height measurements, can refer to the reviews [8,9]. Particularly for the aim of forest height growth inventories, a series of representative studies based on multi-temporal ALS surveys have been carried out [10–15]. These endeavors have concluded that ALS can characterize forest height growths spanning more than two years [12]. At the same time, many tree-level features have been involved, and even the feature of maximum laser height for each tree was deduced as the optimal parameter for forest height growth inventorying [16]. However, these works [10–14] were still plot-wise. That is, height growth cannot be directly measured for each single tree using ALS, even though height growth of individual dominant trees has been reported [15]. In practice, these two limitations may restrict ALS from precise measurements of tree height growths in many applications. To solve this problem, solely improving the sampling densities of ALS systems is not a sound choice. New ideas, at least new variants of laser scanning modes, are preferred.

A large amount of new terrestrial laser scanning modes have been attempted in the last decade. Two typical kinds are static terrestrial laser scanning (TLS) and mobile laser scanning (MLS), and both can generally perform with high sampling densities. With this advantage, TLS has been widely applied for acquiring forest properties in the fine scales [17–20]. For instance, the average error of vertical canopy profiles retrieved from TLS sideway measurements is 4%, much lower than 7% from ALS top-down samplings and 25% from optical hemispherical bottom-up imaging [21]. With stable performance in surveying, TLS even has been assumed as a substitute of the in-field measurement means in more and more application cases [18–20].

With better efficiency than TLS, MLS serves as a state-of-the-art survey technology [22]. The status of MLS systems and techniques can refer to the associated technical reviews [23,24]. The ranges of MLS applications have also kept being enlarged. Other than for infrastructure modeling [25], MLS has also been used for tree-related information processing, e.g., 3D tree modeling with alpha shape [26] and tree modeling within 2D scan profiles [27]. Moreover, a lot of MLS systems supported by diverse platforms have been enthusiastically developed in the last decade, and they can be selectively utilized to measure tree height growths in different environments. For instances, MLS systems onboard sport utility vehicles (SUV) can cope with most plain forests. The boat-based mobile mapping system can be used for river-sided forest monitoring [28]. The pedal-powered tricycles equipped with laser scanners and attitude/position devices can survey urban trees for such as construction of the Google Maps [24]. The sled-borne MLS has been attempted for forest investigations during winter [29]. For the dense or mountainous forests, the Sensei has been designed with hybrid functions of working as mobile and airborne laser scanning, which switch up to different platforms [30]. Overall, the quick developments of MLS and TLS techniques both have manifested potentials for improving the accuracy of tree height growth measurement.

Based on literature review, it seems that the above-mentioned three kinds of laser scanning modes can be combined to improve the performance of tree height growth measurement. The cost-effective frame can be figured out as follows: First, ALS surveys the tree-covered area of interest, and this is aimed at constructing the basic database of tree height distributions. Then, MLS measures several strips of the same area later, and their height differences compared to the corresponding ALS data can be used to derive the associated tree height growth models. At the same time, TLS measures sample trees to calibrate the deduced models. Finally, tree height growths across the whole target area can be imputed. To fulfill this solution frame, the effectiveness of the integration of these three laser scanning modes must be in prior verified. Hence, the objective of this study is to testify the feasibility of using single-scan ALS, TLS and MLS to improve the accuracies of tree height growth measurements. Note that single-scan is emphasized here in favor of the cost-effective demands in practice. Before testing the whole objective, two sub-questions need to be answered: (1) Can this new technique characterize the height growth of each single tree? (2) Can this technique refine the minimum growth-discernable temporal interval further less than two years? The following works will be expanded aimed at the two specific puzzles and the objective of this study.

## 2. Materials

### 2.1. Test Site

The test site is located in a rectangular block of the Espoonlahti region, Southern Finland (60°15′N, 24°65′E). This area has served as the experimental field for evaluating the contributions of multiple cooperative projects. 58 broadleaved Nordic maples (*Crimson King*, *Acer platanoides*) were selected as the sample trees for numerical analysis. These trees behave with obvious morphological changes during annual growth and foliation. In addition, the sample trees stand upright without crown overlaps between each other, and thus, the impacts of laser obscurations between trees can be disregarded during MLS and TLS data collections. The statistical characteristics of the samples are briefly listed in Table 1, wherein the quantitative indices roughly imply that the young trees still maintain high growth speed. Overall, this test site is theoretically appropriate for observing the process of tree height growth.

### 2.2. Data Collection

The campaigns for data collection were deployed in six separate days, entirely spanning three years. First, the ALS survey was conducted using a Topeye MK-II scanner (Topeye, Helsinki, Finland) on 18 December 2006. This day was chosen according to the inference that ALS-based single tree inventory generally performs better in terms of tree height during the leaf-off phases [30]. Next, the TLS survey was carried out with the Roamer [32] in its stop-and-go mode on 7 May 2009, when the maple trees in the boreal regions grew only with tiny buds. Then, the MLS survey based on the same Roamer was run on 10 June 2009, when the sample trees already grew with flourished foliages. Synchronously, twice MLS surveys based on the Sensei [33] were carried out on 6 May 2009 and 8 June 2009 respectively. Finally, another MLS survey based on a Riegl VMX-250 scanner (Riegl, Horn, Austria) was deployed on 21 March 2010, when the sample trees had no leaves emerging yet. The settings of the experimental campaigns, particularly regarding the configurations of all of the laser scanning systems, are listed in Table 2. In the datasets, the Roamer-based TLS (RoT) data and the Riegl-based MLS (RiM) data are used as the references thanks to their high georeferencing accuracies and high sampling densities.

### 2.3. Data Preprocessing

After data collection, any two datasets cannot be directly overlaid to explore tree height growths by differencing tree heights. The reason is that the inconsistency of their initial parameter specifications, e.g., different precisions of their altitude/position modules, different shadowing effects and different scanning geometry are unavoidable. Thus, the first step of data preprocessing was to put all of the independently-georeferenced point clouds together for registration. Here, the registration was fulfilled as follows. The control points were manually picked up from the feature points surrounding the target trees, and then, the rotation matrix and shift vector were solved. The detailed solution was to calculate the associated six parameters for rigid body transformation by means of the iterative closest point algorithm [34]. Accordingly, all of the datasets were registered into the TLS-related coordinate space. This plan took the merits of TLS into account, *i.e.*, TLS generally exhibits higher precisions and stability than MLS and ALS. Next, the 58 sample trees can be isolated for height growth analysis.

**Figure 1.**
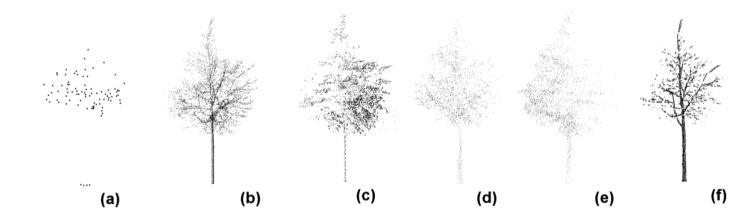
Illustration of the point clusters pertinent to a same tree that is surveyed by laser scanning in multi-modes: (**a**) ToA, (**b**) RiM, (**c**) RoM, (**d**) SeMM, (**e**) SeMJ, and (**f**) RoT.

Automatic algorithms for isolation of single trees in scattered point clouds have been developed in regard to the ALS mode [35] or the MLS mode [36]. However, not all-prevalent methods are available so far. This study attempted to use the commercial TerraScan software (Terrasolid, Helsinki, Finland) to interactively segment the tree-related point clusters. Specifically, the “fence” tool in the TerraScan was used to accomplish this task. First, the point cluster corresponding to a single tree was artificially identified. Next, a close fence was deployed around it. The close fence intuitively works as a cylinder, whose wall surrounds all of the points of interest inside and whose principal axis is collinear with the line of sight. Geometrically, it defines a local space like a cylinder but without ends. In consequence, a target point cluster in three dimensions needs to be restricted by the intersection of three such cylinders with orthogonal principal axes. Finally, the points out of the intersection were excluded and the point cluster corresponding to each individual tree was achieved, as illustrated in Figure 1. In followings, all of the resultant point clusters from the real laser surveys would be investigated to validate the proposed methodologies.

## 3. Methodologies

### 3.1. Analysis of Tree-Top Scan Principles

**Figure 2.**
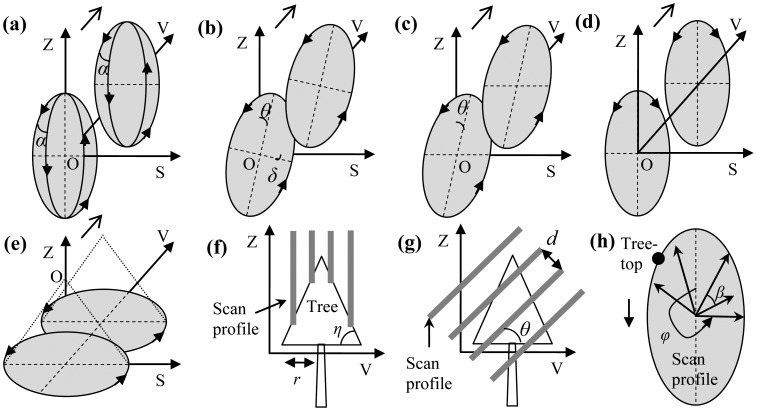
Schematic diagrams of laser scanning mechanisms ((**a**) RoT, (**b**) RiM, (**c**) RoM, (**d**) SeMM and SeMJ, and (**e**) ToA) and schematic diagrams of tree-top scan principles ((**f**) vertical scan profiles and (**g**) tilt scan profiles), wherein (f) features (a), (d) and (e), while (g) refers to (b) and (c). In addition, (**h**) shows the style of laser emissions in a scan profile. O indicates the locations of the laser scanners. See text for the definition of symbols.

Generally with high sampling densities, TLS and MLS tend to represent tree tops more steadily than ALS. Accordingly, the heights of the highest laser hits amidst the point clusters were directly deemed as the heights of the corresponding target trees. This strategy is evidenced by the previous derivations [16], wherein forest height growth estimations were best obtained by employing the tree-level feature of the highest ALS echo height differences, compared to the height differences of height percentiles and digital surface models. However, absolute success of tree-top capturing still cannot be ensured by current TLS and MLS systems. Moreover, different laser scanning systems may perform with different errors on this task. Thus, the tree-top scan principles of all of the referred laser scanning systems were preliminarily analyzed, and the associated schematic diagrams are shown in Figure 2. ALS works in Palmer scan mode. MLS does sampling with parallel scan profiles, as indicated by the bold lines in the Figure 2(f,g). Different MLS systems are configured with different scanning angles (Figure 2(b–d)). TLS operates in a two-axis scanning mode. That is, its vertical scan profile gradually turns with a little angle, after its emitter/receiver completes a circle scanning in the scan profile. The success ratio of tree-top capturing is theoretically up to its location compared to the adjacent scan profiles (Figure 2(f,g)) and the adjacent laser beams (Figure 2(h)).

### 3.2. Estimation of Tree-Top Capturing Errors

The substantial goal of the analyses of tree-top scan principles was to pre-estimate the error ranges of the associated laser-derived tree heights. This is instructive for choosing appropriate laser scanning systems and for predicting tree height growths. In Figure 2, the primary factors impacting laser-based tree-top capturing have been explicitly marked out. The spacing (*d*) between two adjacent scan profiles and the angle (*β*) between two adjacent laser beams in a scan profile are the principal factors. For TLS, the first element can be substituted with the angle (*α*) between two adjacent scan profiles. In addition, the tilt angles (*θ* and *δ*) of scan profiles, the base angle (*η*) and the radius (*r*) of each tree crown, and the field-of-view (*φ*) within scan profiles need to be taken into account. The relevant specifications of the used laser scanning systems are listed in Table 3, wherein

(1) $$d_{1}=2r\cdot \cos\left(\alpha /2\right)$$

(2) $$e_{1}=d\cdot \tan\left(\eta \right)/2$$

(3) $$e_{2}=d/\cos\left(\theta \right)$$

and their ranges of errors can be roughly estimated. It can be further derived that a same laser scanning system in the mode with tilt scan profiles tends to performs better than in the mode with vertical scan profiles, since tree tops are easily omitted in the latter scenario. TLS works more steadily than MLS does, because MLS systems are easily impacted by the accelerations of their vehicles. Collectively, the best configurations of terrestrial laser scanners for stable tree-top capturing can be briefly prescribed as e.g., in the mode with tilt scan profiles and also with the *α* and *β* angles and the spacing *d* as small as possible. Note that the real error factors are more than the variables involved in the error estimation Equations (1)–(3). For example, the hypothesis that a laser beam is always backscattered by a branch in its transmission way cannot be ensured in practice.

### 3.3. Statistics of Tree Height Growths

The estimation of tree-top capturing errors as above indicates that the case of underestimating tree heights is inevitable, even in TLS surveys. Theoretically, this situation has given a negative answer to the first question posed in Section 1. That is, tree-level features do not mean accurate tree-level height growth measurements. Of course, the confirmative answer needs to be concluded from the test results later. Anyhow, the planned approach for investigation of tree height growths was to assume statistical analysis accordingly. In detail, the average height variations regarding a certain number of trees were calculated to characterize their height growths, and this can overcome the errors of laser scanning to some extent. Histogram was employed to depict of the frequency distributions of tree height growths. Gaussian regression was deployed on the histogram to characterize the average height growths, and the fitting was calculated in accordance to

(4) $$f\left(x\right)=a\cdot e^{-{\left(\frac{x-\mu }{\sigma }\right)}^{2}}$$

wherein *x* is the height growth of each tree, *μ* indicates the mean height growth (G_m_), and *σ* denotes the standard deviation of the height growths. Then, the resulting average height growth values can be applied for forest height growth prediction across more extensive areas.

### 3.4. Tree Height and Height Growth Relationship Analysis

The statistical methods used for tree height growth characterization are helpful from the perspective of overcoming the inevitable tree-top capturing errors, but do not mean everything. ALS tended to underestimate tree heights [9,12,31], as illustrated in Figure 1 in this work. Can this survey technique reflect tree height growths effectively? This puzzle requires a further exploitation of the underlying relationships between heights and height growths of the same trees for each kind of the used laser scanning techniques.

**Figure 3.**
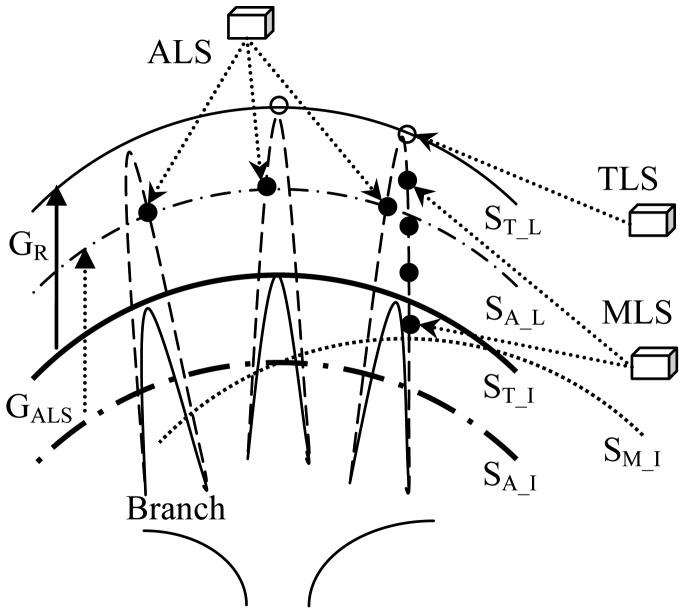
Schematic relationships between the measurements of tree heights and height growths in different laser scanning modes. See text for the definition of symbols.

Figure 3 shows the basic schematics of tree height and height growth characterizations in different laser scanning patterns. In principle, tree heights measured by laser scanning can be represented by the associated canopy surfaces. This simplification stands, since for the deciduous tree species, tree growth performs as the elongations of a bound of branches. Correspondingly, height growths can be solved as the height differences of the two related surfaces. It is worth mentioning that the height differences of canopy surfaces reconstructed from ALS data (G_ALS_ = S_A_L_ − S_A_I_, wherein S means the height of surface, the subscript A_I refers to the initial height measured by ALS, and the subscript A_L notes the height measured by ALS at last) can equally embody the real height growths (G_R_), although tree tops are mostly missed by this scanning mode. This presents the theoretical basis for ALS-based height growth predictions in the previous works [10–14]. The derivation is same for MLS and TLS. The relationship is instructive for constructing the basic rules to judge the correctness of measurements by different laser scanning separately.

### 3.5. Relative Revision of Tree Height Derivation

The above-derived relationships between tree heights and height growths have biases, when height growths are achieved by differentiating two tree heights derived from different kinds of laser scanning. For instance, the sum of the ALS-derived average height and the experience-dependent annual height growth is unnecessarily equal to the TLS-inferred average height in the next year. This suggests that it is requisite to further exploit the underlying relationships between tree heights and height growths of the same trees surveyed by different modes of laser scanning. In view of the goal frame for integration of laser scanning emphasized in this study, the exploitation is equivalently to explore the relationships between tree heights surveyed by TLS, MLS, and ALS at the same time. Then, the above-mentioned biases can be relatively sought and revised. Specifically, according to the indications in Figure 3, two biases need to be tackled. The first bias is the height gap between the TLS-derived surface (S_T_I_) and the ALS-derived surface (S_A_I_), and the second bias is the height gap between the TLS-derived surface (S_T_I_) and the MLS-derived surface (S_M_I_). Regression analysis by linear fitting is used here to exploit the underlying relationships between tree heights derived from different laser scanning data. The fitted formula can be employed to make revisions of tree height derivations from ALS and MLS relative to TLS, and then, the accuracies of tree height growth calculations can be improved.

## 4. Results and Discussions

### 4.1. Growth over Year Interval

#### 4.1.1. Reference Data Acquired by Terrestrial Laser Scanning—One Year Interval

**Figure 4.**
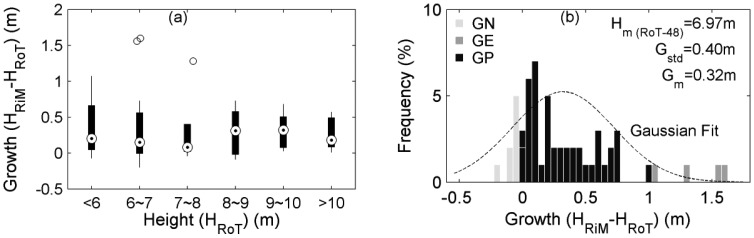
Boxplots (**a**) and histogram (**b**) of the reference one-year height growths derived from the RoT and RiM data collections. See text for the definition of symbols.

The reference height growths over one year were derived from the RoT and RiM data. In order to maintain the conditions consistent with the following comparisons, 48 trees also covered by ToA were considered. After statistics, the distributions of height growths compared to the related tree heights are displayed in the boxplots (Figure 4(a)). It shows that the height growths of these young maple trees all behave in a similar way. Then, the frequencies of tree height growths lying in different height divisions are displayed in the histogram (Figure 4(b)). After Gaussian regression, it is found that the average height growth (G_m_) is 0.32 m, the standard deviation of height growth (G_std_) is 0.40 m, and the mean height pertaining to the surveying day of 7 May 2009 (H_m (RoT-48)_) is 6.97 m. In Figure 4(b), there are several growth-negative (GN) cases, *i.e.*, the heights derived from the survey in the later phase are less than the values pertinent to the initial phase. Here, the later phase corresponds to the RiM survey, and the initial phase refers to the RoT survey. There are also growth-excess (GE) cases, *i.e.*, the heights from the survey in the later phase are excessively larger than the values pertaining to the initial phase, even intuitively out of the reasonable range. Of course, the major cases marked by the growth-positive (GP) are in normal ranges. In fact, the GE and GN cases have verified the theoretically negative answer to the first question in Subsection 3.3. After all, the existence of GE cases shows that single-scan TLS misses tree-tops sometimes and is unreliable for sensing height growth at single tree level.

#### 4.1.2. Integrating Static Terrestrial and Airborne Laser Scanning—Two Years Interval

**Figure 5.**
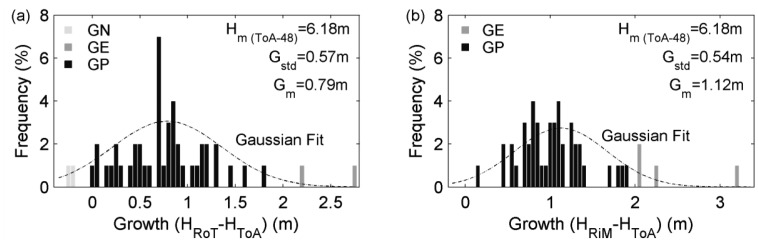
Histogram of the height growths derived (**a**) from the ToA and RoT samplings spanning two years and (**b**) from the ToA and RiM surveys spanning three years. See text for the definition of symbols.

The investigation of tree height growths over two years was deployed on the ToA and RoT surveys, and the latter was used as the reference data. The same 48 trees as mentioned above were processed. The frequencies of tree height growths in multiple height divisions are demonstrated in the histogram (Figure 5(a)). There are also GN and GE cases identified. After Gaussian regression, it is learnt that the average height growth is 0.79 m, and the mean height peculiarly pertaining to the survey day of 18 December 2006 (H_m (ToA-48)_) is 6.18 m. It can be further realized that the annual growths of these same trees are inconsistent, compared to the reference data. Namely, the derived average height growth over two years in this case is larger than the twice of the average height growth over one year retrieved in the reference case. The cause is that the ALS-derived canopy surfaces are overall lower than the real ones. In other words, ALS tends to result in tree height underestimations. This is evidenced by the results that the H_RoT_ is less than the H_ToA_ to some extent. Specifically, those GN cases are the samples that are definitely underestimated.

#### 4.1.3. Integrating Mobile and Airborne Laser Scanning—Three Years Interval

The investigation of tree height growths over three years was executed on the ToA and RiM surveys, and the latter was used as the reference data. The same 48 trees were again involved in statistics. The relevant frequencies of tree height growths in multiple height divisions are likewise manifested in the histogram (Figure 5(b)). Here, there are only GE cases. This is due to that the height growths of these trees over three years are beyond the range of ALS underestimations of tree-tops. After Gaussian regression, it is derived that the average height and mean height growth are 6.18 m and 1.12 m respectively. Compared to the reference results, their mean height growths are inconsistent, similar with the situation pertaining to Figure 5(a). The reason is also same, *i.e.*, the ALS-derived canopy surfaces are generally lower than the real ones. But, the difference of the two average height growths in Figure 5(b) and Figure 5(a) is equal to the reference height growth shown in Figure 4(b). This somehow has testified the presumption that ALS can equivalently characterize tree height growths, although tree tops are easily missed in this scanning mode.

### 4.2. Growth over Month Interval

The Subsection 4.1 has primarily validated the integration of ALS, TLS and MLS for measurement of tree height growths spanning two or three years. However, the year-level interval is unnecessarily the minimum growth-discernable temporal span for this integrated survey technique. It is noticed that the reference data corresponds to the one-year height growths. So, whether the integration of terrestrial laser scanning can distinguish height growths in shorter periods is worth exploring further.

#### 4.2.1. Terrestrial Laser Scanning with High Sampling Densities—One Month Interval

**Figure 6.**
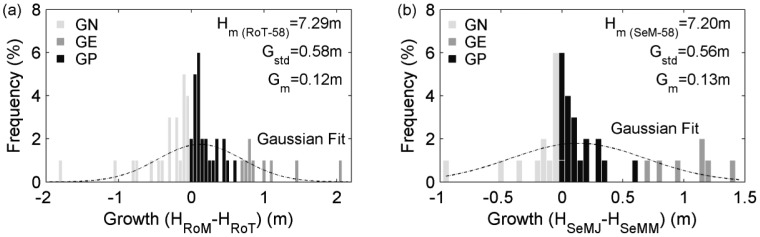
Histogram of the one-month height growths derived (**a**) from the RoT and RoM surveys and (**b**) from the SeMM and SeMJ surveys. See text for the definition of symbols.

The investigation of tree height growths over one month was implemented on the RoM and RoT surveys, and the latter was used as the reference data. All of the 58 trees were involved in statistics. The related frequencies of tree height growths in multiple height divisions are manifested in the histogram (Figure 6(a)). After Gaussian regression, the mean growth is 0.12 m. This value fits the experience that maples in boreal regions grow only for two to three months, and its most thriving growth period in each year is from mid-May to mid-June [37]. The mean height (H_m (RoT-58)_) is 7.29 m, which is different from the opposite value in Figure 4. The proportions of GN and GE cases both increase. This is reasonable due to the reduction of the investigation interval. Additionally, the whole performance gets worse, and this is caused by the fact that the tree-top missing phenomena increase in the RoM surveys. Specifically, the erroneous height measurements happen mostly in the phase of foliages flourishing, when laser obscurations occur more often.

#### 4.2.2. Mobile Laser Scanning with Medium Sampling Densities—One Month Interval

The investigation of tree height growths over one month was also conducted by twice using Sensei, which features the medium sampling densities. The same 58 trees were regarded. The frequencies of tree height growths in different height divisions are demonstrated in the histogram (Figure 6(b)). After Gaussian regression, it is obtained that the mean height is 7.20 m, which is less than the corresponding value derived from the Roamer data. This is due to that Sensei with medium sampling densities tends to miss tree-tops more often than Roamer. In addition, the average height growth is 0.13 m, approximate with the inferred height growth from the Roamer data. This suggests that the MLS systems with medium sampling densities also can equally represent tree height growths, similarly with the scenario of ALS-based growth investigation. In view of the results in this Subsection, it can be derived that by utilizing terrestrial laser scanning systems even with medium sampling densities, the basic growth-discernable temporal interval for tree height growth measurements can also be refined into one month.

### 4.3. Rules for Relative Revision

#### 4.3.1. Mobile Laser Scanning

**Figure 7.**
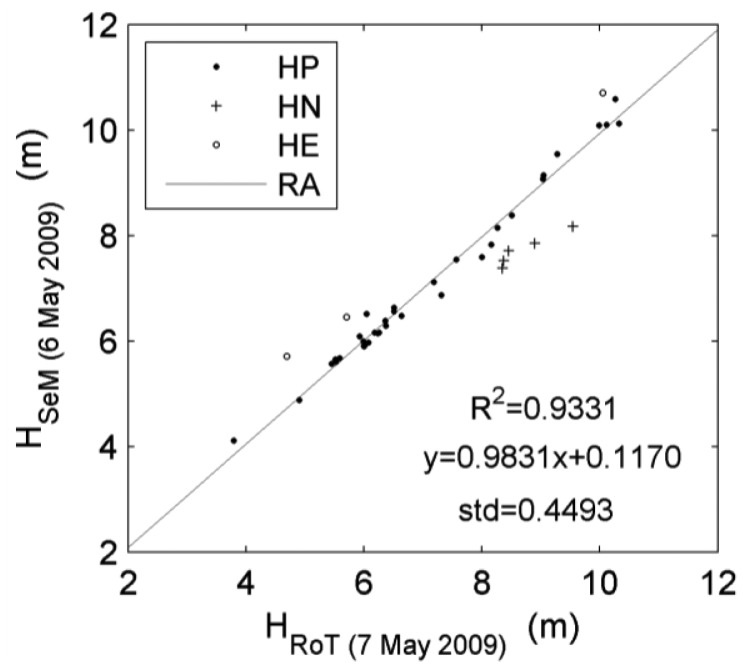
Scatterplots of the tree heights derived from the SeMM and RoT data. See text for the definition of symbols.

The first case of seeking the quantitative rules for relative revision of tree height derivations refers to the SeMM data. The reference data is the point set collected almost synchronously by Roamer in TLS mode on 7 May 2009. The relationships of height pairs are displayed by scatterplots (Figure 7). The linear regression analysis (RA) can supply their fitting line, and the derived linear equation can be deemed as the rule in a simplified form y = 0.9831x + 0.1170 for relative revision. In Figure 7, there are the height-negative (HN), height-positive (HP) and height-excess (HE) pairs. The definitions of these height pairs are different from the GN, GP and GE cases in terms of survey phases, since these height pairs are measured almost simultaneously. Here, the HN and HE pairs are defined according to their distances from the fitted line. If the height values as the response variables are far lower than the fitted line, the related height pairs are defined as HN. If the heights as the response variables are far higher than the fitted line, the related height pairs are defined as HE. The normal range is restricted using the standard deviation. Within the normal range, the height pairs are defined as HP. Although there are the HN and HE phenomena, the high R^2^ value indicates that the Roamer and Sensei surveys are positively correlated in this case. Then, this function can be used to revise the tree heights in other plots in the same Sensei campaign.

#### 4.3.2. Airborne Laser Scanning

**Figure 8.**
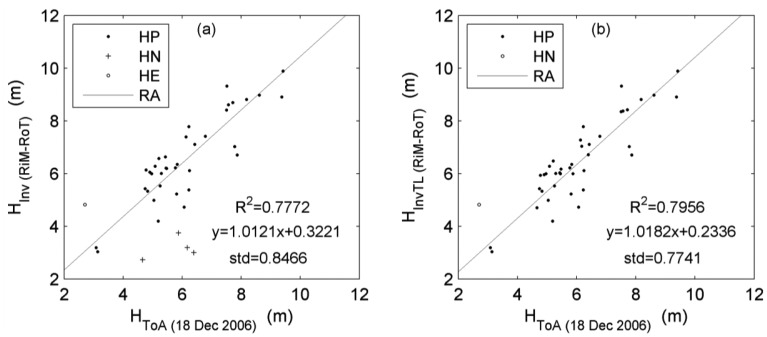
Scatterplots of the tree heights derived from the ToA data and the tree heights inversely derived by combining the RoT and RiM data before relative revision (**a**) and after relative revision (**b**). See text for the definition of symbols.

The second case involves the ALS Topeye MK-II scanner with a low sampling density. The related reference data was generated by using the point sets collected by Roamer in TLS mode on 7 May 2009 and by Riegl in MLS mode on 21 March 2010, since their accuracies are approximate to the accuracies of traditional tree height measurement equipments like clinometers. For each tree, its reference height was acquired by subtracting the twice of the difference between the RiM- and RoT-derived heights from the RoT-derived height. The scatterplots in Figure 8(a) show the relationships of the ALS-derived heights and the reference heights (denoted as H_Inv (RiM-RoT)_). According to the definitions of GE and GN, the calculations of reference heights here can be corrected by pre-cancelling the GE and GN samples in the step of differencing the RiM- and RoT-derived heights. The scatterplots after this correction are shown in Figure 8(b), with R^2^ and standard deviation improved both. The related linear fitting function as the simplified rule can be utilized to relatively revise the tree heights derived from the ALS data in other stands. With the state-of-the-art full waveform laser scanner [38] introduced, the correlation will be further improved. Overall, the two cases above have further validated the effectiveness of the plan of integrating ALS, TLS and MLS for the objective.

## 5. Conclusion

The evaluation shows that the height growth of each individual tree cannot be reliably characterized even by terrestrial laser scanning in the single-scan mode. Note that the limitation of single-scan is highlighted particularly in this study in favor of the demands of cost-effectiveness in practice. Height growths yet need to be assessed by measuring a certain number of sample trees. After statistics, the minimum temporal interval for single-scan terrestrial laser scanning to distinguish tree height growths can be refined into one month, and the related mean height growths can still be discerned. Moreover, it shows that the incorporation of terrestrial laser scanning modes can help revising the parameter of tree height generally underestimated by airborne laser scanning and even mobile laser scanning. The joint usage of the three categories of laser scanning modes can enhance the conventional airborne laser scanning approaches in terms of efficiency, accuracy and adaptability. Overall, the feasibility of the proposed new technique for characterizing tree height growths has been primarily validated.

## Figures and Tables

**Table 1.** Descriptive statistics of the 58 sample trees for test in terms of tree height.

	**H_(7 May 2009) by TLS_ (m)**	**H_(21 Mar 2010) by MLS_ (m)**
Min	3.36	4.08
Max	10.32	10.73
Mean	7.29	7.61
Std	1.81	1.83

**Table 2.** The settings of the campaigns for test and the configurations of all of the pulsed laser scanning systems (time-of-flight (TF) and phase-shift (PS)) for data collection.

**Laser Scanning System**	**Laser Scanner**	**Scan mode (Abbr.)** *	**Range max (m)**	**Pulse mode**	**Wave (nm)**	**Hits /tree**
Topeye MK-II	Topeye MK-II	ALS (ToA)	960	TF	1064	83
Roamer	Faro Photon 80	TLS (RoT)	76	PS	785	7937
Roamer	Faro Photon 80	MLS (RoM)	76	PS	785	9441
Sensei	Ibeo Lux	MLS (SeMM)	200	TF	905	2020
Sensei	Ibeo Lux	MLS (SeMJ)	200	TF	905	2272
Riegl VMX-250	Riegl VQ-250	MLS (RiM)	500	TF	1550	9532

* Note that the abbreviations for short ((MLS system name, e.g., Ro) & (Scan mode, e.g., T)) apply throughout the paper.

**Table 3.** Parametric specifications of the used laser scanning systems and the related tree-top capturing error estimations.

	***θ* (°)**	***δ* (°)**	***α* (°)**	***β* (°)**	***φ* (°)**	***d* (m)**	**Error Estimation (m)**
ToA	90	0	-	-	360	0.3	*e*_1_
RiM	45	45	-	0.12	360	0.04	*e*_2_
RoM	45	0	-	0.096	360	0.17	*e*_2_
SeM	0	0	-	0.25	110	0.4	*e*_1_
RoT	0	0	0.15	0.125	360	*d*_1_	*e*_1_
